# Striatal Dopamine D_2_-Muscarinic Acetylcholine M_1_ Receptor–Receptor Interaction in a Model of Movement Disorders

**DOI:** 10.3389/fphar.2020.00194

**Published:** 2020-03-13

**Authors:** René A. J. Crans, Elise Wouters, Marta Valle-León, Jaume Taura, Caio M. Massari, Víctor Fernández-Dueñas, Christophe P. Stove, Francisco Ciruela

**Affiliations:** ^1^Laboratory of Toxicology, Department of Bioanalysis, Ghent University, Ghent, Belgium; ^2^Unitat de Farmacologia, Departament Patologia i Terapèutica Experimental, Facultat de Medicina, IDIBELL-Universitat de Barcelona, L’Hospitalet de Llobregat, Barcelona, Spain; ^3^Institut de Neurociències, Universitat de Barcelona, Barcelona, Spain; ^4^Programa de Poìs-graduação em Bioquiìmica, Centro de Ciencias Bioloìgicas, Universidade Federal de Santa Catarina, Florianoìpolis, Brazil

**Keywords:** D_2_R, M_1_R, sumanirole, VU0255035, striatum, Parkinson’s disease

## Abstract

Parkinson’s disease (PD) is a neurodegenerative disorder characterized by motor control deficits, which is associated with the loss of striatal dopaminergic neurons from the substantia nigra. In parallel to dopaminergic denervation, there is an increase of acetylcholine within the striatum, resulting in a striatal dopaminergic–cholinergic neurotransmission imbalance. Currently, available PD pharmacotherapy (e.g., prodopaminergic drugs) does not reinstate the altered dopaminergic–cholinergic balance. In addition, it can eventually elicit cholinergic-related adverse effects. Here, we investigated the interplay between dopaminergic and cholinergic systems by assessing the physical and functional interaction of dopamine D_2_ and muscarinic acetylcholine M_1_ receptors (D_2_R and M_1_R, respectively), both expressed at striatopallidal medium spiny neurons. First, we provided evidence for the existence of D_2_R–M_1_R complexes via biochemical (i.e., co-immunoprecipitation) and biophysical (i.e., BRET^1^ and NanoBiT^®^) assays, performed in transiently transfected HEK293T cells. Subsequently, a D_2_R–M_1_R co-distribution in the mouse striatum was observed through double-immunofluorescence staining and AlphaLISA^®^ immunoassay. Finally, we evaluated the functional interplay between both receptors via behavioral studies, by implementing the classical acute reserpine pharmacological animal model of experimental parkinsonism. Reserpinized mice were administered with a D_2_R-selective agonist (sumanirole) and/or an M_1_R-selective antagonist (VU0255035), and alterations in PD-related behavioral tasks (i.e., locomotor activity) were evaluated. Importantly, VU0255035 (10 mg/kg) potentiated the antiparkinsonian-like effects (i.e., increased locomotor activity and decreased catalepsy) of an ineffective sumanirole dose (3 mg/kg). Altogether, our data suggest the existence of putative striatal D_2_R/M_1_R heteromers, which might be a relevant target to manage PD motor impairments with fewer adverse effects.

## Introduction

Parkinson’s disease (PD) is a common movement disorder that is clinically characterized by motor control deficits, such as bradykinesia, muscular rigidity, resting tremors, and postural instability (Mhyre et al., 2012). Approximately, 1% of the population older than 60 years is affected by PD. The major pathophysiological PD hallmark is the loss of dopaminergic neurons projecting from the substantia nigra pars compacta (Hisahara and Shimohama, 2011; Dexter and Jenner, 2013), which leads to dopamine (DA) depletion within the striatum. L-3,4-dihydroxyphenylalanine (L-DOPA) is an effective DA replacement strategy, which efficiently reverses motor control deficits at the early stages of the disorder. However, long-term L-DOPA therapy (>5–10 years) is commonly associated with adverse motor complications, such as dyskinesia and efficacy fluctuations, thus reducing the patient’s quality of life (Jenner, 2003; Kalia and Lang, 2015). Currently, DA receptor agonists (i.e., pramipexole and ropinirole) are considered the first choice in PD therapy, as monotherapy or adjuvants to L-DOPA (Fox et al., 2011; Fox et al., 2018). Again, these agonists are effective at the early stages, but they eventually fail reducing motor complications (Jenner, 2003; Hisahara and Shimohama, 2011). Interestingly, before L-DOPA was extensively prescribed, anticholinergics were the first-line therapeutics in PD (Carlsson et al., 1957; Katzenschlager et al., 2003). The cholinergic system plays a pivotal role in regulating striatal functions by modulating the excitability of GABAergic medium spiny neurons (MSNs), which constitute nearly 95% of the striatal neuronal population (Lv et al., 2017). Nowadays, anticholinergics (i.e., biperiden) are eventually used as adjuvant drugs in PD management, besides their adverse effects (i.e., nausea, cognitive impairments, dry mouth, urinary retention, and blurred vision). Importantly, some of these adverse effects are likely due to a lack of muscarinic acetylcholine receptor (mAChR) subtype selectivity, because both M_2_R and M_3_R are blocked (Chen and Swope, 2007; Pedrosa and Timmermann, 2013). Recently, cholinergic modulation of striatal functions has gained renewed interest because of the development of compounds targeting specific mAChR subtypes (Xiang et al., 2012; Shen et al., 2015; Ztaou et al., 2016; Lv et al., 2017; Chambers et al., 2019).

Five distinct mAChR subtypes (M_1_R–M_5_R) have been identified, which are classified into two groups, based on pharmacological and molecular characteristics. The excitatory M_1_-like receptors (M_1_R, M_3_R, and M_5_R) transduce their signals via G_*q/*__11_ proteins, whereas the inhibitory M_2_-like receptors (M_2_R and M_4_R) are coupled to G_*i/o*_ proteins (Zhang et al., 2002; Bordia and Perez, 2019). All subtypes are present in the striatum, with M_1_R and M_4_R being highly expressed and modulating the excitability of GABAergic MSNs (Hersch et al., 1994; Yan et al., 2001). In general, two types of MSNs have been distinguished: (i) dopamine D_2_ receptors (D_2_Rs) expressing MSNs (i.e., D_2_R-MSNs), which belong to the striatal indirect pathway (Gagnon et al., 2017); and (ii) dopamine D_1_ receptors (D_1_Rs) containing MSNs (i.e., D_1_R-MSNs) constituting the striatal direct pathway. The D_1_R-MSNs express postsynaptic M_4_Rs, whereas M_1_Rs are expressed by both D_1_R-MSNs and D_2_R-MSNs. Thereby, within the striatum, tonically active cholinergic interneurons (ChIs), which constitute 1% to 2% of the total striatal neuronal population (Bolam et al., 1984; Pisani et al., 2007), release acetylcholine (ACh) through widely arborizing axons with large terminal fields that modulate the MSNs via M_1_Rs and M_4_Rs (Graybiel, 1990; Mesulam et al., 1992; Contant et al., 1996). Interestingly, the modulation of MSNs with a selective M_1_R antagonist resulted in antiparkinsonian-like effects in a number of rat models of movement disorders (Xiang et al., 2012). In addition, the blockade of M_1_R, M_4_R, or ChI signaling improved the motor functions in 6-hydroxydopamine–lesioned mice (Ztaou et al., 2016). Furthermore, systemic administration of scopolamine (a non-selective mAChR antagonist) modulated the DA turnover and reduced D_2_R affinity of raclopride in monkey brains (Tsukada et al., 2000). These studies suggest an intense neuronal interaction between dopaminergic and cholinergic systems, where normal motor functions may require a fine-tuned and coordinated control (Di Chiara et al., 1994; Calabresi et al., 2000; Zhang et al., 2002). The extent to which both neurotransmission systems specifically integrate at a molecular and/or functional level is of high interest for the development of novel multimodal pharmacological therapies to manage PD (Fuxe et al., 2012).

Here, we describe a novel interaction between the D_2_R and M_1_R in the striatum, which may eventually harmonize with those previously described for D_2_R (Cabello et al., 2009; Lukasiewicz et al., 2010; Borroto-Escuela et al., 2013; Bonaventura et al., 2014; Fernandez-Duenas et al., 2015; Rico et al., 2017; Vasudevan et al., 2019). In addition, we evaluated the antiparkinsonian efficacy of a combined D_2_R agonist (i.e., sumanirole) and M_1_R antagonist (i.e., VU0255035) treatment using the reserpine animal model of experimental parkinsonism. To our knowledge, this study is the first to demonstrate a molecular interaction and a functional interplay between D_2_R and M_1_R.

## Materials and Methods

### Plasmid Construction

The plasmids pFLAG-D_2_R, pHA-M_1_R, pD_2_R-*R*luc, pM_1_R-YFP, and pEYFP were a kind gift of Dr. Kjell Fuxe (Karolinska Institutet, Stockholm, Sweden). The sequence encoding the human M_1_R (NM_000738.3) was polymerase chain reaction–amplified using primers containing specific restriction sites (*Hin*dIII and *Eco*RI, 5′-GCTTAAGCTTATGAACACTTCAG-3′ and 5′- TCGAGAATTCGCGCATTGGC-3′) and cloned into the *Hin*dIII/*Eco*RI sites of the NanoBiT^®^ vector NB MCS1 (Promega, Madison, WI, United States). The construct was verified by restriction digest and Sanger sequencing (Eurofins Genomics, Ebersberg, Germany). This resulted in the fusion of the split NanoLuciferase (NL) fragment LargeBiT (LgBiT; 18 kDa) to the C-terminus of M_1_R. The constructs of cannabinoid types 1 and 2 receptors (CB_1_R and CB_2_R, respectively) and D_2_R fused with LgBiT or Small BiT (SmBiT; 1 kDa) were previously developed and described by our research group (Cannaert et al., 2016; Wouters et al., 2019a).

### Cell Culture and Transient Transfection

Human embryonic kidney 293T (HEK293T; American Type Culture Collection, Manassas, VA, United States) cells were maintained in Dulbecco modified Eagle medium (DMEM; Thermo Fisher Scientific, Pittsburg, PA, United States) supplemented with GlutaMAX, 10% fetal bovine serum (FBS; Merck KgaA, Darmstadt, Germany), streptomycin (100 μg/mL), and penicillin (100 μ/mL) in a controlled environment (37°C, 98% humidity, and 5% CO_2_). Prior to transfection, cells were cultured in 10-cm dishes (co-immunoprecipitation) or six-well plates [Bioluminescence Resonance Energy Transfer^1^ (BRET^1^) and NanoLuciferase Binary Technology (NanoBiT^®^) assays] in 10 or 2 mL DMEM supplemented with 10% FBS, respectively. The HEK293T cells were transiently transfected using the polyethylenimine (Sigma-Aldrich, St. Louis, MO, United States) method. In all assays, medium was refreshed with DMEM + 10% FBS after 5 h.

### Co-immunoprecipitation

HEK293T cells were transfected with 5 μg of the constructs containing pFLAG-D_2_R and/or pHA-M_1_R. When necessary, 5 μg of the empty vector pcDNA3.1 was co-transfected to maintain a total amount of 10 μg DNA per 10-cm dish. After 48 h, cells were washed three times with ice-cold phosphate-buffered saline (PBS; 1.47 mM KH_2_PO_4_, 8.07 mM Na_2_HPO_4_, 137 mM NaCl, 0.27 mM KCl with pH 7.2), harvested, and centrifuged, after which the pellet was stored at −80°C until further use. The cells were homogenized in ice-cold 50 mM Tris–HCl (pH 7.4) with the Polytron at setting six for two periods of 10 s. Subsequently, the homogenates were transferred to 1.5-mL Eppendorf and centrifuged at 12,000 × *g* for 30 min at 4°C. Then, all supernatant was removed, and the pellets were lysed in radioimmunoprecipitation assay (RIPA) buffer [150 mM NaCl, 25 mM Tris–HCl (pH 7.5), 1% sodium deoxycholate, 1% NP-40, and 0.1% sodium dodecyl sulfate (SDS)], supplemented with freshly added protease inhibitors (2.5 g/mL aprotinin, 1 mM PEFA-block, 10 g/mL leupeptin), for 1 h while rotating at 4°C. The samples were centrifuged at 12,000 × *g* for 20 min at 4°C. Next, the supernatant of each sample was transferred to a new Eppendorf, and the protein concentrations were determined using the bicinchoninic acid (BCA) assay (Pierce Biotechnology, Rockford, IL, United States). Thereafter, all samples were diluted with RIPA buffer to obtain equal protein concentrations with a final volume of 500 μL. An amount of 10% for each sample (i.e., lysate) was denatured at 37°C for 10 min in 4 × Laemmli [5% SDS, 50% glycerol, 65 mM Tris–HCl (pH 6.8) and 0.2% bromophenol blue], supplemented with freshly added 10% β-mercaptoethanol. The lysates were loaded onto a 10% polyacrylamide 10-well gel and resolved via SDS–polyacrylamide gel electrophoresis (SDS-PAGE). Subsequently, the proteins were blotted onto a nitrocellulose membrane (Amersham Protran 0.45 NC; GE Healthcare Life Sciences, Freiburg, Germany) and subjected to immunoblot analysis, as described below. The other 90% of each sample [i.e., immunoprecipitates (IPs)] was used for immunoprecipitation through adding 2 μg mouse anti-FLAG antibody (clone M2; Sigma-Aldrich) or mouse anti-HA antibody (clone 16B12; Abcam, Cambridge, United Kingdom). After 1.5 h of rotation, 20 μL of washed immobilized Protein-A UltraLink^®^ Resin (#53139; Thermo Fisher Scientific) was added to the IPs, and the rotation continued for 1.5 h at 4°C. Then, the beads were washed three times with RIPA buffer supplemented with the freshly added protease inhibitors. The proteins were eluted and denatured from the beads by heating the samples for 10 min at 37°C in RIPA buffer and 4 × Laemmli supplemented with freshly added 10% β-mercaptoethanol. All IP eluates were subjected to SDS-PAGE electrophoresis and immunoblotting, as described above.

Immunoblots containing lysates or IPs were blocked in PBS with Licor blocking buffer (1:1; LI-COR Biosciences, Lincoln, NE, United States) at room temperature (RT) for 1 h. Then, the immunoblots were incubated with rabbit anti-HA (1:2,000, #GTX29110; Genetex, Irvine, CA, United States) or rabbit anti-FLAG (1:1000, #PA1-984B; Thermo Fisher Scientific) antibodies in 1:1 Licor blocking buffer-PBST (PBS with 0.05% Tween 20) overnight at 4°C. The blots were washed three times with PBST for 10 min at RT. Next, the blots were incubated with donkey anti–rabbit secondary antibodies (1:15,000), conjugated to IRDye680RD or IRDye800CW (LI-COR Biosciences), for 1 h at RT. After incubation, the blots were washed three times with PBST and two times with PBS, each for 10 min at RT, protected from the light. Protein bands were visualized by the Odyssey imaging system (LI-COR Biosciences).

### Bioluminescence Resonance Energy Transfer^1^ Assay

HEK293T cells were transfected with a constant amount of pD_2_R-*R*luc (200 ng) and increasing amounts of pM_1_R-YFP or pEYFP (0–1,000 ng). Equal DNA ratios were maintained with co-transfection of the empty vector pcDNA3.1, which equilibrated the total amount of transfected DNA. Forty-eight hours posttransfection, the cells were washed three times with PBS, detached, and resuspended in Hanks balanced salt solution (HBSS; Thermo Fisher Scientific). An aliquot was used to determine the protein concentrations via the BCA assay, to control the number of cells. All cell suspensions were diluted to a density corresponding to a final protein concentration of 600 ng/μL. Cell suspensions (corresponding to 20 μg protein) were distributed in duplicates into white and black 96-well microplates (#3600 and #3650; Corning, Stockholm, Sweden) for BRET^1^ and fluorescence measurements, respectively. The substrate, *h*-coelenterazine (Molecular Probes, Eugene, OR, United States), was added at a 5 μM final concentration. After 1 min (BRET^1^) and 10 min (*R*luc total), the signals were measured using the ClarioSTAR microplate reader (BMG Labtech, Ortenberg, Germany) through the sequential integration of signal detection at 475 nm (445–505 nm) and 530 nm (500–560 nm). The net BRET^1^ ratio was expressed as a ratio of the light intensity at 530 nm over 475 nm by subtracting the background signal, which was detected when D_2_R-*R*luc was only expressed with pcDNA3.1. The BRET^1^ curve was obtained by fitting the data points to a non-linear regression equation assuming a single binding site using GraphPad Prism version 6.00 (San Diego, CA, United States).

### NanoLuciferase Binary Technology^®^ Assay

HEK293T cells were transfected with constructs encoding for pM_1_R-LgBiT (200 ng) and pD_2_R-SmBiT (200 ng). As negative controls, the cells were transfected with a combination of pM_1_R-LgBiT and pCB_1_R-SmBiT or pD_2_R-SmBiT and pCB_2_R-LgBiT, each with DNA concentrations of 200 ng. The functionality of the CB_1_R-SmBiT and CB_2_R-LgBiT constructs was demonstrated before (Cannaert et al., 2016). In all conditions, the construct encoding for the fluorescent protein Venus was co-transfected (5% of the total DNA transfected). The NanoBiT^®^ assay was performed as described previously (Wouters et al., 2019a). Briefly, 48 h posttransfection, the cells were washed two times with PBS, detached, and centrifuged for 5 min at 1,000*g* at RT. Protein concentrations were determined on an aliquot via the BCA assay, and cell suspensions, normalized for cell number (via a corresponding protein concentration of 600 ng/μL), were diluted in HBSS. Following a 20-fold dilution of the Nano-Glo^®^ Live Cell reagent (#N2011; Promega) containing the luminescent substrate furimazine in aqueous Nano-Glo LCS dilution buffer, 25 μL of the diluted substrate was added to the wells of a 96-well plate containing 100 μL cell suspension. Fluorescence (508–548 nm) or luminescence (440–480 nm) emission was measured with the ClarioSTAR microplate reader in black or white 96-well plates (#3650 and #3600; Corning), respectively. The luminescence data were normalized for the measured fluorescence signals to avoid signal fluctuations due to variations in transfection efficiencies.

### Animals

Caesarean derived 1 (CD-1) mice (Janvier Labs, Le Genest-Saint-Isle, France), D_2_R knockout (D_2_R KO) CD-1, and M_1_R knockout (M_1_R KO) C57BL/6J mice were generated as described previously (Fisahn et al., 2002; Taura et al., 2017). Animals were housed and tested in compliance with the guidelines described in the Guide for the Care and Use of Laboratory Animals (Clark et al., 1997) and following the European Communities Council Directive (2010/63/EU), FELASA, and ARRIVE guidelines. The animals were conventionally housed in groups of four or five in a temperature-controlled (22°C) and humidity-controlled (66%) environment under a 12-/12-h light–dark cycle, where food and water intake was *ad libitum*. The study protocol was approved by the Ethical Committee on Animal Use and Care of the University of Barcelona (CEEA/UB). All efforts were made to minimize animal suffering and the number of animals used in this study. Behavioral tests were performed with wild-type (WT) mice aged 5 months, weighing 40 to 55 g, between 12:00 and 18:00.

### Double Immunofluorescence Staining

M_1_R KO mice were kindly provided by Dr. Adrian James Mogg (Eli Lilly and Company Ltd., Windlesham, United Kingdom) with permission of Dr. Jurgen Wess {National Institute of Diabetes and Digestive and Kidney Diseases, National Institutes of Health (NIH), Bethesda, MD, United States]. These mice were anesthetized and perfused intracardially with 50 to 200 mL of ice-cold 4% formaldehyde solution (Sigma-Aldrich) in PBS. Subsequently, the brains were postfixed in 4% formaldehyde solution overnight at 4°C. D_2_R KO and WT littermate fixed mouse brains were a kind gift from Dr. Jean-Martin Beaulieu (Centre de recherche en Santé Mentale de Québec, Québec, QC, Canada). Coronal brain sections (50 μm) were made with the Vibratome 1200S (Leica Lasertechnik GmbH, Heidelberg, Germany). Finally, the slices were collected and stored in antifreeze solution (30% glycerol, 30% ethylene glycol in PBS with pH 7.2) at -20°C until further processing. The coronal brain slices of WT, D_2_R KO, and M_1_R KO mice were washed three times with PBS and permeabilized with 0.3% Triton X-100 in PBS for 2 h at RT. Then, blocking was performed by incubating the slices with washing solution (PBS with 0.05% Triton X-100) containing 5% normal donkey serum (NDS; Jackson ImmunoResearch Laboratories, Inc., West Grove, PA, United States) for 2 h at RT. Subsequently, the slices were incubated overnight at 4°C with rabbit anti-M_1_R polyclonal (1:300, #mAChR-M1-Rb-Af340; Frontier Institute Co., Ltd., Shinko-nishi, Ishikari, Hokkaido, Japan) and guinea pig anti-D_2_R polyclonal (1:300, #D2R-GP-Af500; Frontier Institute Co., Ltd.) antibodies in washing solution with 1% NDS. In parallel, overnight incubations of WT brain slices only in washing solution served as additional negative controls. After overnight incubation, the slices were washed three times with washing solution containing 1% NDS for 10 min at RT. Next, slices were incubated with Alexa Fluor^®^ 488–conjugated donkey anti–guinea pig (1:400, #706-545-148; Jackson ImmunoResearch Laboratories) and Cy3-conjugated donkey anti–rabbit (1:400, #711-166-152, Jackson ImmunoResearch Laboratories) antibodies in washing solution with 1% NDS for 2 h at RT. Then, the slices were washed three times with washing solution for 10 min at RT and stained with 4,6-diamidino-2-phenylindole (DAPI; 1 μg/mL, #D9542; Sigma-Aldrich) for 15 min at RT. Finally, slices were washed twice with washing solution, twice with PBS for 10 min at RT, and preserved in Vectashield (#H-1000; Vector Laboratories, Burlingame, CA, United States). Images were captured with a Zeiss laser scanning microscope 880 (Carl Zeiss AG, Jena, Germany).

### AlphaLISA^®^ Immunoassay

The AlphaLISA^®^ immunoassay was performed as previously described (Fernandez-Duenas et al., 2019). Briefly, WT and D_2_R KO animals were euthanized by cervical dislocation, followed by dissection of striata on an ice-cold plate. Then, striatum was rapidly homogenized in ice-cold 50 mM Tris–HCl (pH 7.4) with a Polytron at setting six for three periods of 10 s. The homogenate was centrifuged at 1,000 × *g* for 10 min, and the supernatant was transferred to a new Eppendorf. The protein concentrations were determined with the BCA assay, and the membrane fractions were centrifuged at 12,000 × *g* for 30 min. The pellets were resuspended in assay buffer [20 mM MgCl_2_, 130 mM NaCl, 0.2 mM EDTA, 0.1 mg/mL saponin, and 0.5% immunoglobulin G (IgG)–free bovine serum albumin] to a final protein concentration of 1.5 μg/μL. Donkey anti–guinea pig IgGs (#706-005-148; Jackson ImmunoResearch Laboratories) were conjugated to the acceptor beads (#6762001; Perkin Elmer, Waltham, MA, United States), according to the manufacturer’s instructions. Subsequently, 10 μL of each striatal membrane in assay buffer was distributed in triplicate into a white 384-well plate (384 Well Small Volume HiBase Microplates; Greiner Bio-one, Kremsmünster, Austria) and stored for 1 h at 4°C. Subsequently, the membranes were incubated with rabbit anti-M_1_R polyclonal (10 nM, #mAChR-M1-Rb-Af340; Frontier Institute Co., Ltd.) and guinea pig anti-D_2_R polyclonal (10 nM, #D2R-GP-Af500; Frontier Institute Co., Ltd.) antibodies in assay buffer overnight at 4°C. In the WT-negative controls, only the anti-M_1_R antibody was added, whereas the D_2_R KO–negative controls were incubated with assay buffer overnight at 4°C. Next, acceptor beads (40 μg/mL) were added to each well for 1 h. Then, the anti–rabbit IgG alpha donor beads (40 μg/mL, #AS105D; Perkin Elmer) were added and mixed with the acceptor beads by pipetting up and down. Any prolonged light exposure was avoided. Finally, after 1-h incubation, the donor beads were excited (640–720 nm), and acceptor beads emission (597–633 nm) was measured with the ClarioSTAR microplate reader.

### Locomotor Activity Tests

Mice were administered subcutaneously (s.c.) with reserpine (3 mg/kg; Sigma-Aldrich) or vehicle (saline with 5% Tween 20, s.c.) 20.5 ± 2 h before the test. Then, mice were administered with vehicle [saline with 5% dimethyl sulfoxide (DMSO) and 5% Tween 20, i.p.], sumanirole (1, 3, or 10 mg/kg, i.p.; Sigma-Aldrich) and/or VU0255035 (10 mg/kg, i.p.; Tocris Biosciences, Bristol, United Kingdom) 10 min before each locomotor activity test. The mice were evaluated for drug-induced locomotor activity as described previously (Taura et al., 2017). Briefly, non-habituated mice were placed in the center of an activity field apparatus (30 × 30 cm, surrounded by four 50-cm-high black walls) equipped with a camera above to record activity. Exploratory behavior of the animals was recorded for 85 min. The distance traveled was analyzed using the Spot tracker function from ImageJ (NIH). All locomotor activity tests were performed in a sound attenuated room, illuminated by light of 15 lux. After each trail, the apparatus was cleaned with 70% alcohol and rinsed with water.

### Horizontal Bar Test

Catalepsy was induced in mice by the administration of reserpine (3 mg/kg, s.c.) overnight (20.5 ± 2 h). Vehicle, sumanirole (1, 3, or 10 mg/kg, i.p.) and/or VU0255035 (10 mg/kg, i.p.) was administered, and 1.5 h later, catalepsy was measured as described previously (Massari et al., 2017; Taura et al., 2017). Briefly, using a stopwatch with a cutoff time of 120 s, the duration of an abnormal upright posture was measured, in which the forepaws of the mouse were placed on a horizontal wooden bar (0.6-cm diameter) that was located 4.5 cm above the floor.

### Tremulous Jaw Movements

Mice were administered reserpine (3 mg/kg, s.c.) or vehicle (saline with 5% Tween 20, s.c.). Subsequently, vehicle (saline with 5% DMSO and 5% Tween 20, i.p.), sumanirole (1-10 mg/kg, i.p.), and/or VU0255035 (10 mg/kg, i.p.) was administered 1.5 h before the test and 22 ± 2 h after reserpine treatment. The tremulous jaw movements (TJMs) were measured with hand-operated counters, as described previously (Massari et al., 2017). Briefly, the mice were placed individually in a glass cylinder (13-cm diameter) and allowed to habituate for 10 min. Mirrors were placed under and behind the cylinder to allow observation when the animal faced away from the observer. Tremulous jaw movements were defined as rapid vertical deflections of the lower jaw that resembled chewing, but were not directed to any particular stimulus (Salamone et al., 1998). The incidence of these oral movements was measured continuously for 10 min, but were discounted during grooming.

### Statistical Analysis

The number of biological replicates (n) in each experimental condition is indicated in the figure legends. Data of behavioral studies are expressed as the mean ± SEM; all the other data are presented as the mean ± SD. Numerical data were imported to GraphPad Prism version 6.00 for Windows (GraphPad Software, La Jolla, CA, United States). Statistical analysis of cellular or tissue data was performed using the Mann–Whitney *U* test or the non-parametric analysis of variance (ANOVA) by ranks of Kruskal–Wallis test followed by the Dunn multiple-comparisons *post hoc* test. Normal distributions of the behavioral data were inferred through the D’Agostino–Pearson normality test. Subsequently, behavioral data were analyzed with the one-way ANOVA or the two-way repeated-measures ANOVA followed by the Tukey or Dunnett multiple-comparisons *post hoc* test. *p* ≤ 0.05 was considered as statistically significant.

## Results

### D_2_R–M_1_R Interaction in HEK293T Cells

**FIGURE 1:**
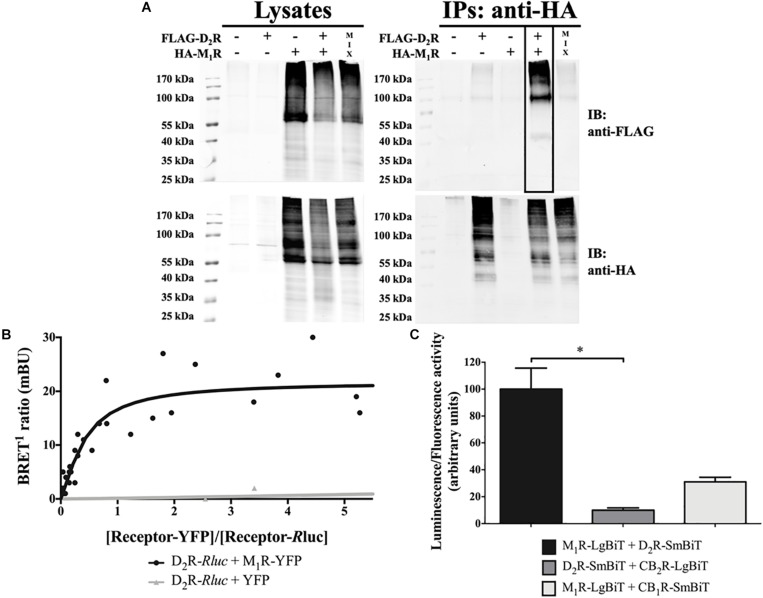
D_2_R–M_1_R interaction in transiently transfected HEK293T cells. **(A)** Co-immunoprecipitation. HEK293T cells were harvested and lysed 48 h after transfection. The lysates were used for immunoblotting (IB) with anti-FLAG and anti-HA antibodies to demonstrate D_2_R and M_1_R expression, respectively (left panels). The rest of the samples (IPs) was subjected to immunoprecipitation with a mouse anti-HA antibody. The co-immunoprecipitate was confirmed via the detection of FLAG-D_2_R upon IB with rabbit anti-FLAG and rabbit anti-HA antibodies (right panel; boxed lane). Data shown are representative of three independent experiments. **(B)** BRET^1^ saturation curve. The BRET^1^ signal in HEK293T cells co-expressing a constant amount of D_2_R-*R*luc and increasing amounts of M_1_R-YFP (*n* = 5) or YFP (*n* = 3) constructs was measured 48 h posttransfection. The BRET^1^ saturation curve is derived from all independent experiments. **(C)** NanoBiT^®^ complementation assay. The SmBiT and LgBiT parts of the NanoLuciferase fragments were fused to the C-terminus of the indicated receptor. The constructs were overexpressed via transient transfection in HEK293T cells. Results are presented as mean ± SD (*n* = 3). Statistical significance was tested using the non-parametric ANOVA by ranks of Kruskal–Wallis followed by the Dunn multiple-comparisons *post hoc* test, *p ≤ 0.05.

The ability of D_2_R and M_1_R to physically interact in living cells was assessed by biochemical and biophysical assays. First, co-immunoprecipitation experiments were performed in transiently transfected HEK293T cells. Interestingly, when HA-M_1_R was immunoprecipitated from FLAG-D_2_R and HA-M_1_R co-transfected HEK293T cells, a specific immunoreactive band of 90 to 100 kDa corresponding to FLAG-D_2_R was detected (Figure 1A, IPs). It is important to note that this band was not observed when the cells were transfected with a single receptor plus an empty plasmid or from an extract mix of separate transfected cells. Moreover, the D_2_R and M_1_R constructs were properly expressed in the whole setup (Figure 1A, lysates). These results indicate that D_2_R and M_1_R are expressed within the same membrane context and are prone to interact.

Subsequently, the existence of D_2_R–M_1_R complexes was verified by means of BRET^1^ saturation assays. Accordingly, HEK293T cells were co-transfected with a constant amount of the D_2_R-*R*luc construct and increasing concentrations of M_1_R-YFP or YFP plasmids (Figure 1B). A positive BRET signal was observed when D_2_R-*R*luc and M_1_R-YFP were co-expressed, due to the energy transfer between *R*luc and YFP. Conversely, in cells co-expressing D_2_R-*R*luc and YFP, no BRET^1^ signal was observed. Overall, the BRET^1^ data demonstrated that D_2_R and M_1_R are in close proximity (<10 nm), thus supporting the existence of D_2_R–M_1_R complexes in living cells (Cottet et al., 2012; Dacres et al., 2012).

Finally, we implemented the complementation-based NanoBiT^®^ assay to further validate the D_2_R–M_1_R interaction in HEK293T cells (Figure 1C). This assay utilizes two inactive fragments of a split NL, which, when fused to two interacting proteins, come into close proximity and reassemble into a functional protein (Wouters et al., 2019b). As shown in Figure 1C, co-expression of M_1_R and D_2_R fused to the large and small subunits of a split NL (M_1_R-LgBiT and D_2_R-SmBiT, respectively) yielded a high luminescent signal (Figure 1C) when compared to HEK293T cells expressing either constructs for M_1_R and CB_1_R (M_1_R-LgBiT + CB_1_R-SmBiT) or D_2_R and CB_2_R (D_2_R-SmBiT + CB_2_R-LgBiT), as previously reported (Wouters et al., 2019a). In addition, very low signals were observed in cells expressing either M_1_R (19 ± 3.5) or D_2_R (9 ± 1.7), along with HaloTag-SmBiT or HaloTag-LgBiT, respectively. Altogether, our results are compatible with the formation of D_2_R/M_1_R heteromer formation by ectopically expressed M_1_R and D_2_R in HEK293T cells.

### Co-distribution of D_2_R and M_1_R in the Mouse Striatum

**FIGURE 2:**
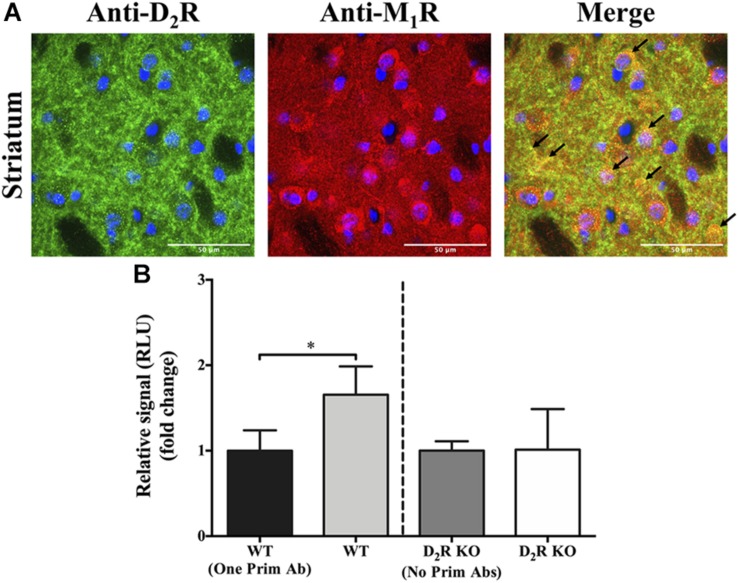
Co-distribution of D_2_R and M_1_R in the mouse striatum. **(A)** Double-immunofluorescence staining. Representative images of specific immunoreactivities with anti-D_2_R and anti-M_1_R antibodies in the dorsal striatum of wild-type (WT) CD-1 mice. Overlapping immunofluorescence signals are indicated with arrows. Images shown are representative of two independent experiments. Scale bar = 50 μm. **(B)** AlphaLISA^®^ immunoassay. Specific signal obtained from striatal WT and D_2_R KO mice, with or without adding one or two primary antibodies. Results are presented as mean ± SD (*n* = 4). Statistical significance was tested using the Mann–Whitney *U* test, *p ≤ 0.05.

Once the existence of D_2_R–M_1_R complexes in a heterologous expressing system was demonstrated, we aimed to verify whether this interaction might also occur in native tissue. To this end, we first analyzed D_2_R and M_1_R expression in mouse striatum by double-immunofluorescence staining. The specificity of the anti-D_2_R and anti-M_1_R antibodies was verified by using striatal slices from D_2_R- and M_1_R-deficient mice (D_2_R KO and M_1_R KO, respectively) (Supplementary Figures S1 and S2). High-magnification images of the dorsal striatum from WT mice showed a high degree of D_2_R and M_1_R co-distribution (Figure 2A, arrows). Subsequently, to further demonstrate a close proximity (<200 nm) between both receptor types, we applied an AlphaLISA^®^ immunoassay, as described previously (Fernandez-Duenas et al., 2019). Briefly, striatal membrane extracts were first incubated with specific primary antibodies against the receptor, which can be recognized by secondary antibodies tagged with beads able to engage in an energy transfer after the production of a singlet oxygen (Fernandez-Duenas et al., 2019). A significant higher energy transfer was observed in the WT compared to its corresponding negative control [WT vs. WT (one Prim Ab); *p* ≤ 0.05, Figure 2B]. In addition, analysis of striatal D_2_R KO tissue did not result in a significant difference in signal with or without adding primary antibodies (Figure 2B). These results support the existence of the interaction (or at least the very close proximity) between D_2_R and M_1_R in native tissue, namely, the mouse striatum.

### Multimodal D_2_R Agonist and M_1_R Antagonist Treatment of Reserpinized Mice

The data obtained in HEK293T cells and striatal slices support the notion that D_2_R and M_1_R might physically interact in the striatum. Therefore, we hypothesized that this receptor–receptor interaction might constitute a molecular target for multimodal pharmacological interventions finely controlling striatal motor activity. Accordingly, we tested the effects of a combined drug treatment regimen (D_2_R agonist + M_1_R antagonist) in a well-known model of movement disorder, i.e., the reserpinized mouse (Leao et al., 2015; Leal et al., 2016). The drugs used were the D_2_R-selective agonist sumanirole and the M_1_R-selective antagonist VU0255035. Sumanirole was chosen as it shows 200-fold more selectivity for D_2_R than for other DA receptors subtypes and as it has been used both in human patients and animal models of PD (McCall et al., 2005; Stephenson et al., 2005; Barone et al., 2007). Similarly, the competitive orthosteric antagonist VU0255035 has a 75-fold higher selectivity for M_1_R over other mAChR subtypes (Sheffler et al., 2009). In addition, both compounds have already been tested individually in reserpine-treated animals (McCall et al., 2005; Xiang et al., 2012).

First, we evaluated the effects of the D_2_R agonist sumanirole. Mice were treated with reserpine (3 mg/kg, s.c., overnight) and, thereafter, with the selective D_2_R agonist. Interestingly, sumanirole only promoted an increase in locomotion at the highest dose (10 mg/kg) (Supplementary Figure S3). Similarly, only at 10 mg/kg, sumanirole blocked the cataleptic effects induced by reserpine, while a slight but non-significant reduction of TJMs was observed (Supplementary Figure 3). Thus, based on these data, we selected 3 mg/kg of sumanirole (i.e., subthreshold dose) for further multimodal experiments in combination with the M_1_R antagonist VU0255035. A dosage of 10 mg/kg of VU0255035 was selected based both on a pilot study and its pharmacokinetic profile. According to Sheffler et al. (2009), 10 mg/kg VU0255035 (i.p.) was sufficient to cross the blood-brain barrier, with maximal M_1_R inhibition after 30 min, with an elimination half-life of ∼2.5 h in the brain. In addition, this concentration was also reported to not impair contextual fear conditioning, a model for hippocampus-dependent learning (Sheffler et al., 2009).

**FIGURE 3:**
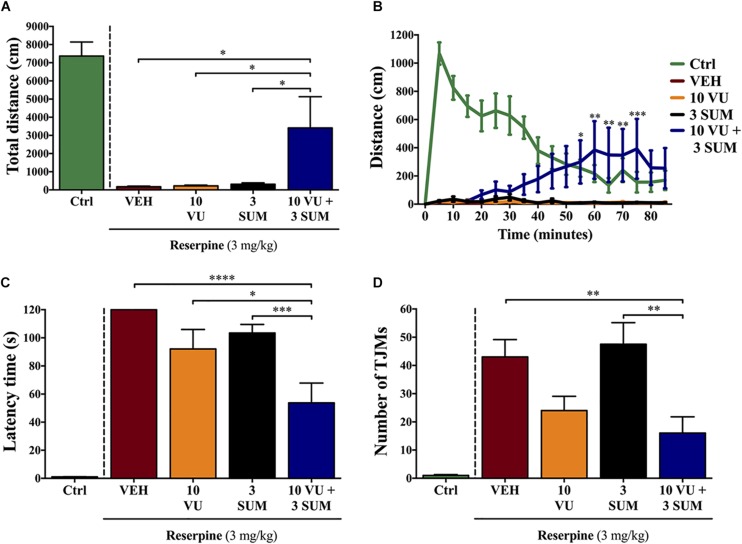
Effect of the combinatorial treatment of suboptimal dosages of sumanirole and VU0255035 on reserpine-induced motor disturbances in mice. Mice treated with saline (control mice = Ctrl), VEH (saline with 5% Tween, i.p.), VU (VU0255035, 10 mg/kg, i.p.), SUM (sumanirole, 3 mg/kg. i.p.), or VU + SUM (VU0255035, 10 mg/kg and sumanirole, 3 mg/kg, i.p.) after reserpine administration (3 mg/kg, s.c., 20.5 ± 2 h) were evaluated via the **(A,B)** locomotor activity test, **(C)** horizontal bar test, and **(D)** for tremulous jaw movements (TJMs). **(A)** The total distance traveled (cm) was measured for 85 min. Results are presented as mean ± SEM (*n* = 8–9 animals). Statistical significance was tested using one-way ANOVA followed by the Dunnett *post hoc* test with VEH, VU, and SUM compared to VU + SUM animals, *p ≤ 0.05. **(B)** The distance traveled (cm) was measured every 5 min for 85 min. Results are presented as mean ± SEM (*n* = 8–9 animals). Statistical significance was tested using two-way repeated-measures ANOVA followed by the Tukey *post hoc* test with VEH, VU and SUM compared to VU + SUM animals, *p ≤ 0.05, **p ≤ 0.01, and ***p ≤ 0.001. **(C)** Reserpine-induced catalepsy in mice evaluated via the horizontal bar test with a cutoff value of 120 s. Results are presented as mean ± SEM (*n* = 8–13 animals). Statistical significance was tested using one-way ANOVA followed by the Tukey *post hoc* test with VEH, VU, and SUM compared to VU + SUM animals, *p ≤ 0.05, ***p ≤ 0.001, and ****p ≤ 0.0001. **(D)** Reserpine-induced orofacial dyskinesia evaluated by TJM frequency for 10 min. Results are presented as mean ± SEM (*n* = 9–13 animals). Statistical significance was tested using one-way ANOVA followed by the Tukey *post hoc* test with VEH, VU, and SUM compared to VU + SUM animals, **p ≤ 0.01.

In animals that received the combined treatment with VU0255035 and sumanirole (VU + SUM; 10 and 3 mg/kg, i.p.) we observed a significant (*p* ≤ 0.05) reversal of the reserpine-induced akinesia (Figure 3). In contrast, in none of the animals treated with VU or SUM alone the akinetic status was reversed (Figure 3A). Our findings suggest a fine balance in locomotor activity between reserpine-induced akinesia and VU + SUM treatment. It is interesting to note that the VU + SUM–administered animals showed an increase in locomotor activity after ∼25 min, which is in accordance with the pharmacokinetic profile of VU (Figure 3B). Thus, significant differences in locomotion were observed between the VU + SUM–treated group compared to the groups receiving a single treatment. In line with the results obtained while evaluating locomotion, a significant reduction in reserpine-induced catalepsy was observed in VU + SUM–treated mice compared to those that were administered a single agent (Figure 3C). However, while the simultaneous VU0255035 and sumanirole administration reduced TJMs as compared to vehicle, when compared to single administered animals, no differences were found with VU0255035-treated reserpinized mice (Figure 3D). Therefore, a low dose of sumanirole was unable to potentiate the VU0255035-mediated TJMs reduction. This lack of sumanirole-mediated potentiation of VU0255035 effect might be due to the fact that 10 mg/kg VU0255035 already induced a slight, but not significant (*p* = 0.1712), reduction in TJMs. Of course, it would be reasonable to speculate that M_1_Rs located within neuronal circuits controlling distinct behavioral responses might have different efficacies. Overall, our data support the use of low D_2_R agonist doses in combination with an M_1_R antagonist as a novel multimodal antiparkinsonian pharmacotherapy.

## Discussion

In the last years, G protein-couped receptor (GPCR) oligomers have gained interest as novel putative targets for several diseases. One of the most well-characterized D_2_R-containing oligomers is the D_2_R/A_2__*A*_R heteromer in the striatum, where reciprocal antagonistic interactions both at the binding and effector levels occur between these receptors (Ferré et al., 2018). Importantly, this functional interplay grounded the utility of A_2__*A*_R blockade in PD treatment, which recently ended with the approval of a selective A_2__*A*_R antagonist, istradefylline (Nourianz), as an adjuvant drug in PD treatment. Interestingly, while a variety of D_2_R oligomer complexes has been described (Marcellino et al., 2008; Trifilieff et al., 2011; Bonaventura et al., 2014; Borroto-Escuela et al., 2014; Hasbi et al., 2017), few studies exist for M_1_R (Goin and Nathanson, 2006; Hern et al., 2010). In the present study, we have observed, for the first time, the existence of striatal D_2_R and M_1_R complexes. In addition, we provide data supporting a novel multimodal antiparkinsonian treatment, consisting of the use of low D_2_R agonist doses in combination with M_1_R antagonists. Thus, our results may prompt further investigating these receptor complexes as interesting targets to modulate dopaminergic neurotransmission in dopamine-related diseases (i.e., PD) (Hersch et al., 1994; Surmeier et al., 2007; Fuxe et al., 2012).

As described for the D_2_R/A_2__*A*_R heteromer, both M_1_R and D_2_R are expressed at postsynaptic membranes of striatopallidal MSNs. Thus, the avidity of D_2_R to heteromerize with a named GPCR (i.e., A_2__*A*_R, mGlu_5_R or M_1_R) within this specific subcellular domain may depend on the absolute expression of specific protomers and the relative affinities shown for each receptor–receptor interaction. Importantly, the density of each individual D_2_R containing oligomer may be altered in disease conditions, which may constitute a putative pathological fingerprint. Precisely, we recently reported that D_2_R/A_2__*A*_R heteromers would be increased in the caudate from human postmortem PD patients (Fernandez-Duenas et al., 2019). This fact would negatively affect dopaminergic neurotransmission, thus providing the rationale for using A_2__*A*_R antagonists in PD (see above). Of note, whether the decrease in heteromer formation is a cause or a consequence of PD pathology, or even treatment, needs to be further elucidated. Here, we demonstrated the existence of D_2_R–M_1_R complexes in the striatum and its potential pharmacotherapeutic usefulness using an animal model of PD. However, further studies should be conducted to determine: (i) D_2_R/M_1_R heteromer status in human PD striatum (i.e., increase or decrease in the proportion of D_2_R and M_1_R protomers forming homomers or heteromers) and (ii) the molecular and functional interplay with other striatal D_2_R-containing oligomers (i.e., D_2_R/A_2__*A*_R heteromers). Certainly, establishing the D_2_R-containing heteromer status in PD could determine the design of selective combined pharmacotherapeutic strategies restoring the unbalanced dopaminergic neurotransmission associated with PD.

In our study, the functional interplay between D_2_R and M_1_R was demonstrated by the co-administration of a D_2_R agonist and an M_1_R antagonist to reserpinized mice, which is an animal model mimicking parkinsonian motor and non-motor impairments (Leao et al., 2015; Leal et al., 2016). The major disadvantage of this model is the lack of dopaminergic neurons degeneration and protein aggregation. Nevertheless, reserpine-treated rodents have been successfully applied to predict the efficacy of many dopaminergic and non-dopaminergic drugs (e.g., benzotropine), which are clinically in use for PD management. The high predictive validity of this model results in the maintenance of its position as a valid choice to discover novel therapeutics in an early preclinical stage (Duty and Jenner, 2011). Other advantages are its low toxicity, low cost, and its reproducibility among laboratories (Leao et al., 2015). Furthermore, the reserpine animal model was one of the first models used to demonstrate the therapeutic efficacy of L-DOPA, which still remains the criterion standard in PD therapy (Carlsson et al., 1957). Now, D_2_R selective agonists are also included in the pharmacotherapeutic munition in PD management. Of note, although the full D_2_R agonist sumanirole has a high affinity for D_2_R, it also has a moderate affinity for the serotonin 5-HT_1__*A*_ receptor (K_*i*_ = 95 nM) (Heier et al., 1997; Wuts, 1999; McCall et al., 2005). However, according to Weber et al. (2010), the suboptimal sumanirole concentration applied in our study should not result in 5-HT_1__*A*_ receptor off-target effects (Weber et al., 2010). It is worth mentioning that our results, using a suboptimal concentration of sumanirole, are not in line with the findings of another study, which also used a reserpine animal model (McCall et al., 2005). The discrepancy could be owing to differences in species (mice *vs.* rats), reserpine inductions (3 mg/kg vs. 5 mg/kg + AMPT), administration routes of sumanirole (i.p. vs. s.c.), and/or time of reserpine pretreatments (20.5 vs. 18 h). Nevertheless, a long-term effect in locomotion at high sumanirole doses was demonstrated in both studies, which has been suggested to be the result of postsynaptic D_2_R activation (McCall et al., 2005). On the other hand, the administered dose of the competitive orthosteric M_1_R antagonist VU0255035 results in maximal receptor inhibition, with a high brain penetration after 30 min, without impairment in hippocampus-dependent learning tasks (Sheffler et al., 2009). The combined treatment increased locomotor activity and decreased the time of catalepsy and the amount of TJMs in our animal model, whereas the reduction in TJMs was mostly due to the M_1_R antagonist (Lees, 2005; Pedrosa and Timmermann, 2013).

The dysregulation of dopaminergic or cholinergic systems has been linked to movement disorders, such as dystonia, Huntington disease, or PD (Pisani et al., 2007). Nowadays, at the early stages, PD therapy is commonly initiated with D_2_R agonists, which do not require carrier-mediated transport or produce potentially toxic metabolites and free radicals (Hagan et al., 1997; Jenner, 2003). However, D_2_R agonists may elicit severe adverse effects such as valvular heart disease or psychiatric disturbances (Lees, 2005; Hisahara and Shimohama, 2011; Pedrosa and Timmermann, 2013), which are probably induced by activating D_3_Rs and D_4_Rs (Rich et al., 1995; McCall et al., 2005). Despite its high D_2_R selectivity, sumanirole has not demonstrated a clinical improvement over ropinirole (Barone et al., 2007; Singer et al., 2007). However, as suggested by the present study, sumanirole remains a valuable tool in lead optimization, drug discovery, and animal models, where the novel D_2_R–M_1_R interaction may provide a rationale to target specific receptor subtypes in the treatment of PD. In addition, reducing the amount of D_2_R agonist by supplementing an M_1_R selective antagonist (i.e., VU0255035) in a multimodal pharmacological approach may allow achieving an effective treatment and induce less adverse effects.

Muscarinic acetylcholine receptors play important roles in cognitive, motor, behavioral, sensory, and autonomic processes. Thus, non-selective blockade of mAChRs is associated with important side effects, including cognitive deficits. While scopolamine, a non-selective mAChR antagonist, robustly increased locomotor activity in reserpinized akinetic rats, it induced learning and memory impairments (Sheffler et al., 2009; Xiang et al., 2012). Importantly, most cognitive adverse effects observed with anticholinergic therapies are likely due to the result of M_2_R and M_3_R blockade (Fornari et al., 2000; Wess et al., 2007). Conversely, the selective M_1_R blockade has been shown to exhibit some antiparkinsonian activity, although without the full efficacy as observed with non-selective anticholinergics (Xiang et al., 2012; Lv et al., 2017; Chambers et al., 2019). This is probably due to activation of other mAChRs, which also have important roles in the motor circuits of the basal ganglia (e.g., M_4_R). Indeed, antagonizing M_1_R mainly has an excitatory effect on GABAergic MSNs, but no or only a partial effect at the subthalamic nucleus and substantia nigra pars reticulata (Xiang et al., 2012; Lv et al., 2017). Interestingly, mice lacking M_1_R have increased locomotor activity (Gerber et al., 2001; Miyakawa et al., 2001). These M_1_R KO mice also have increased extracellular dopamine levels in the striatum, which suggests that inhibiting M_1_R positively affects PD treatment (Gerber et al., 2001). Moreover, M_1_R KO mice were shown to maintain contextual fear recognition, which indicates that M_1_R might not be involved in the initial stability of memory or in its formation in the hippocampus (Miyakawa et al., 2001; Anagnostaras et al., 2003). Accordingly, the main benefit to target M_1_R over other mAChRs is due to its selective role in controlling locomotor activity, whereas its input is less critical for cognitive processes (Miyakawa et al., 2001).

In conclusion, here we demonstrated, for the first time, an interaction between D_2_R and M_1_R. Interestingly, our results suggest an extensive integration of dopaminergic and cholinergic neurotransmission systems in the striatum, where inhibition by DA is predicted to facilitate locomotor activity, and activation by ACh inhibits locomotion via striatopallidal MSNs (Di Chiara et al., 1994). Using reserpinized mice as a model, we demonstrated the effectiveness of a multimodal treatment, combining a suboptimal dosage of the selective D_2_R agonist sumanirole and the M_1_R-specific antagonist VU0255035. Overall, further functional exploitation of this novel D_2_R–M_1_R interaction (i.e., identifying the functional fingerprint of this putative new heterodimer in native tissue) may provide beneficial opportunities in PD treatment.

## Data Availability Statement

All datasets generated for this study are included in the article/Supplementary Material.

## Ethics Statement

The animal study was reviewed and approved by the Ethical Committee on Animal Use and Care of the University of Barcelona (CEEA/UB).

## Author Contributions

RC performed and designed the experiments, analyzed the data and wrote the manuscript. EW performed the NanoBiT^®^ assay, MV-L performed the AlphaLISA^®^ assay. JT and CM performed *in vivo* experiments. VF-D designed the experiments and wrote the manuscript. CS supervised the project and wrote the manuscript. FC supervised the project, designed experiments and wrote the manuscript.

## Conflict of Interest

The authors declare that the research was conducted in the absence of any commercial or financial relationships that could be construed as a potential conflict of interest.

## Supplementary Material

FIGURE S1 Validation of the anti-D_2_R and anti-M_1_R antibodies via double immunofluorescence staining in mice brains. Images of coronal slices from mice brains representing the dorsal striatum, corpus callosum and cortex with staining of D_2_R-positive cells (green), M_1_R-positive cells (red) and DAPI-positive nuclei (blue). Minimal signal intensities were observed with the anti-D_2_R and anti-M_1_R antibodies in the D_2_R and M_1_R KO mice, respectively. Data shown are representative of two independent experiments. Scale bar = 100 μm.

FIGURE S2 Validation of the anti-D_2_R and anti-M_1_R antibodies via Western Blotting. **(A)** The anti-D_2_R antibody used in our study demonstrates specificity for D_2_R in striatal tissue. Extracts of the striatum from D_2_R KO, D_2_R heterozygous (HET), and wild-type (WT) CD-1 littermates were loaded on 10% SDS-PAGE. The anti-α-Tubulin antibody was used to control for equal loading of the samples. **(B)** The anti-M_1_R antibody used in the study demonstrates specificity for M_1_R in striatal tissue. Striatal extracts from M_1_R KO and wild-type (WT) with C57BL/6J background were loaded on 10% SDS-PAGE. The anti-α-Tubulin antibody was used to control for equal loading of the samples. kDa = kilodalton.

FIGURE S3 Sumanirole dosage-response of reserpine-induced motor disturbances in mice. The mice were treated with VEH (saline and 5% Tween, i.p.), or 1, 3, or 10 mg/kg SUM (sumanirole, 1, 3, 10 mg/kg, respectively, i.p.) after reserpine administration (3 mg/kg, s.c., 20.5 ± 2 h), and evaluated via the **(A,B)** locomotor activity test, **(C)** horizontal bar test and **(D)** for tremulous jaw movements (TJMs). **(A)** The total distance traveled (cm) was measured for 85 min. Results are presented as mean ± SEM (*n* = 7–8 animals). Statistical significance was tested using one-way ANOVA, followed by the Dunnett *post hoc* test, with VEH, 1 SUM, and 3 SUM compared to 10 SUM animals, ^∗∗^*p* ≤ 0.01. **(B)** The distance traveled (cm) was measured every 5 min for 85 min. Results are presented as mean ± SEM (*n* = 7–8 animals). Statistical significance was tested using two-way repeated-measures ANOVA followed by the Tukey *post hoc* test, with VEH, 1 SUM, and 3 SUM compared to 10 SUM animals, ^∗^*p* ≤ 0.05, ^∗∗^*p* ≤ 0.01, ^∗∗∗^*p* ≤ 0.001 and ^****^*p* ≤ 0.0001. **(C)** Reserpine-induced catalepsy in mice evaluated via the horizontal bar test, with cut-off value of 120 s. Results are presented as mean ± SEM (*n* = 7–8 animals). Statistical significance was tested using one-way ANOVA followed by the Tukey *post hoc* test, ^∗∗^*p* ≤ 0.01. **(D)** Reserpine-induced orofacial dyskinesia evaluated by TJMs for 10 min. Results are presented as mean ± SEM (*n* = 7–8 animals). Statistical significance was tested using one-way ANOVA followed by the Tukey *post hoc* test, ^∗∗^*p* ≤ 0.01.
