# Corrigendum: Striatal dopamine D2-muscarinic acetylcholine M1 receptor-receptor interaction in a model of movement disorders

**DOI:** 10.3389/fphar.2022.1075433

**Published:** 2022-11-18

**Authors:** René A. J. Crans, Elise Wouters, Marta Valle-León, Jaume Taura, Caio M. Massari, Víctor Fernández-Dueñas, Christophe P. Stove, Francisco Ciruela

**Affiliations:** ^1^ Laboratory of Toxicology, Department of Bioanalysis, Ghent University, Ghent, Belgium; ^2^ Unitat de Farmacologia, Departament Patologia i Terapèutica Experimental, Facultat de Medicina, IDIBELL-Universitat de Barcelona, L’Hospitalet de Llobregat, Barcelona, Spain; ^3^ Institut de Neurociències, Universitat de Barcelona, Barcelona, Spain; ^4^ Programa de Poìs-graduação em Bioquiìmica, Centro de Ciencias Bioloìgicas, Universidade Federal de Santa Catarina, Florianoìpolis, Brazil

**Keywords:** D_2_R, M_1_R, sumanirole, VU0255035, striatum, Parkinson’s disease

In the published article, there was an error in Figure 1A as published. The immunoblot panel corresponding to the Lysates/IB: anti-FLAG (upper left corner) was erroneously swapped with the immunoblot panel of IPs: anti-HA/IB: anti-HA (lower right corner). The corrected Figure 1 and its caption appear below.

**FIGURE 1:**
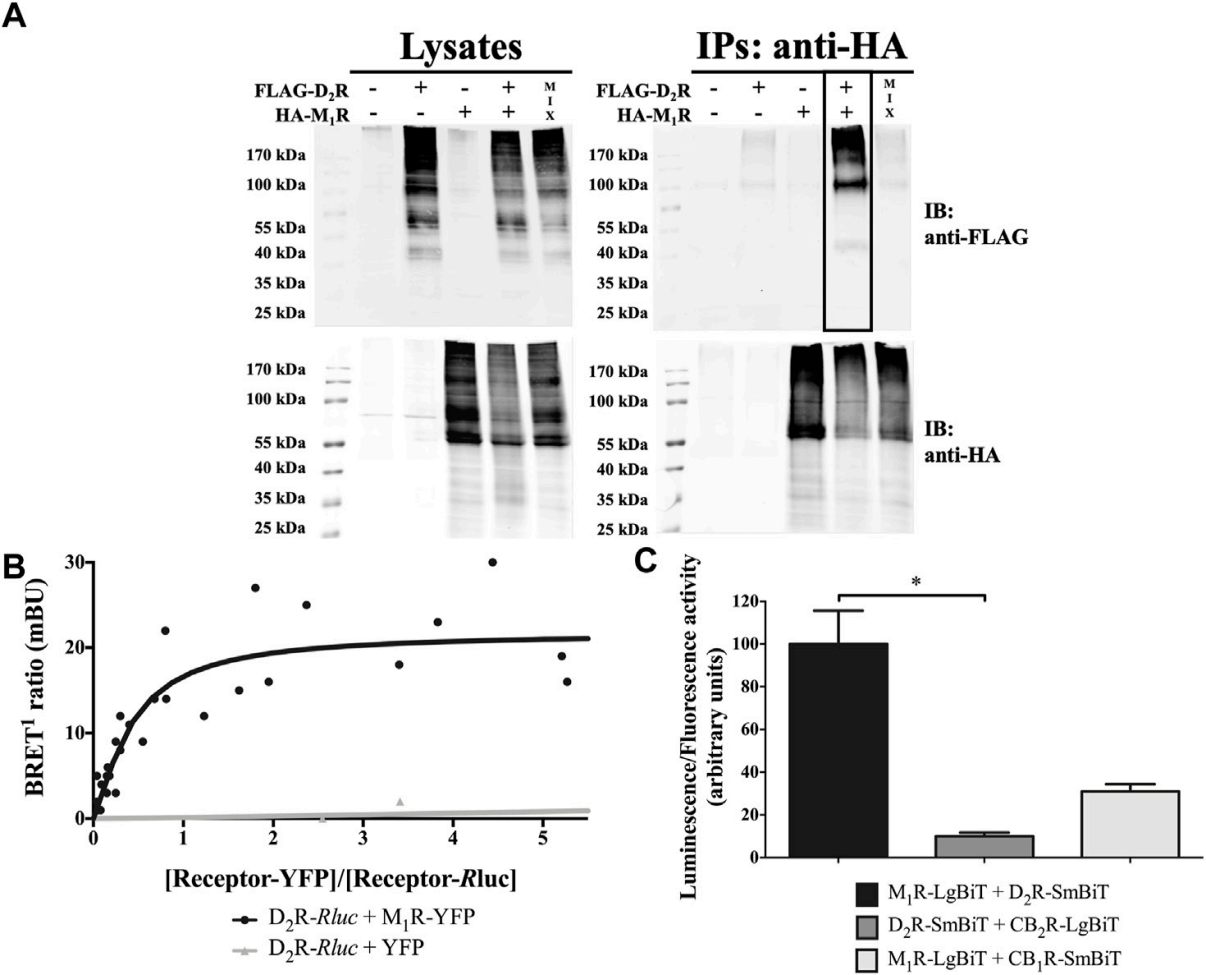
D_2_R-M_1_R interaction in transiently transfected HEK293T cells. **(A)** Co-immunoprecipitation. HEK293T cells were harvested and lysed 48 h after transfection. The lysates were used for immunoblotting (IB) with anti-FLAG and anti-HA antibodies to demonstrate D_2_R and M_1_R expression, respectively (left panels). The rest of the samples (immunoprecipitates; IPs) were subjected to immunoprecipitation with a mouse anti-HA antibody. The CoIP was confirmed *via* the detection of FLAG-D_2_R upon IB with rabbit anti-FLAG and rabbit anti-HA antibodies (right panel; boxed lane). Data shown are representative of three independent experiments. **(B)** BRET^1^ saturation curve. The BRET^1^ signal in HEK293T cells co-expressing a constant amount of D_2_R-*R*luc and increasing amounts of M_1_R-YFP (*n* = 5) or YFP (*n* = 3) constructs was measured 48 h post-transfection. The BRET^1^ saturation curve is derived from all independent experiments. **(C)** NanoBiT^®^ complementation assay. The SmBiT and LgBiT parts of the NanoLuciferase fragments were fused to the C-terminus of the indicated receptor. The constructs were overexpressed *via* transient transfection in HEK293T cells. Results are presented as mean ± SD (*n* = 3). Statistical significance was tested using the nonparametric ANOVA by ranks of Kruskal–Wallis followed by the Dunn’s multiple comparisons *post-hoc* test, * = *p* ≤ 0.05.

The authors apologize for this error and state that this does not change the scientific conclusions of the article in any way. The original article has been updated.

