# Supplementary figures and images for: Striatal Dopamine D2-Muscarinic Acetylcholine M1 Receptor–Receptor Interaction in a Model of Movement Disorders
Source: Front Pharmacol. 2020 Mar 13;11:194. doi: 10.3389/fphar.2020.00194 (PMC7083216; doi:10.3389/fphar.2020.00194)

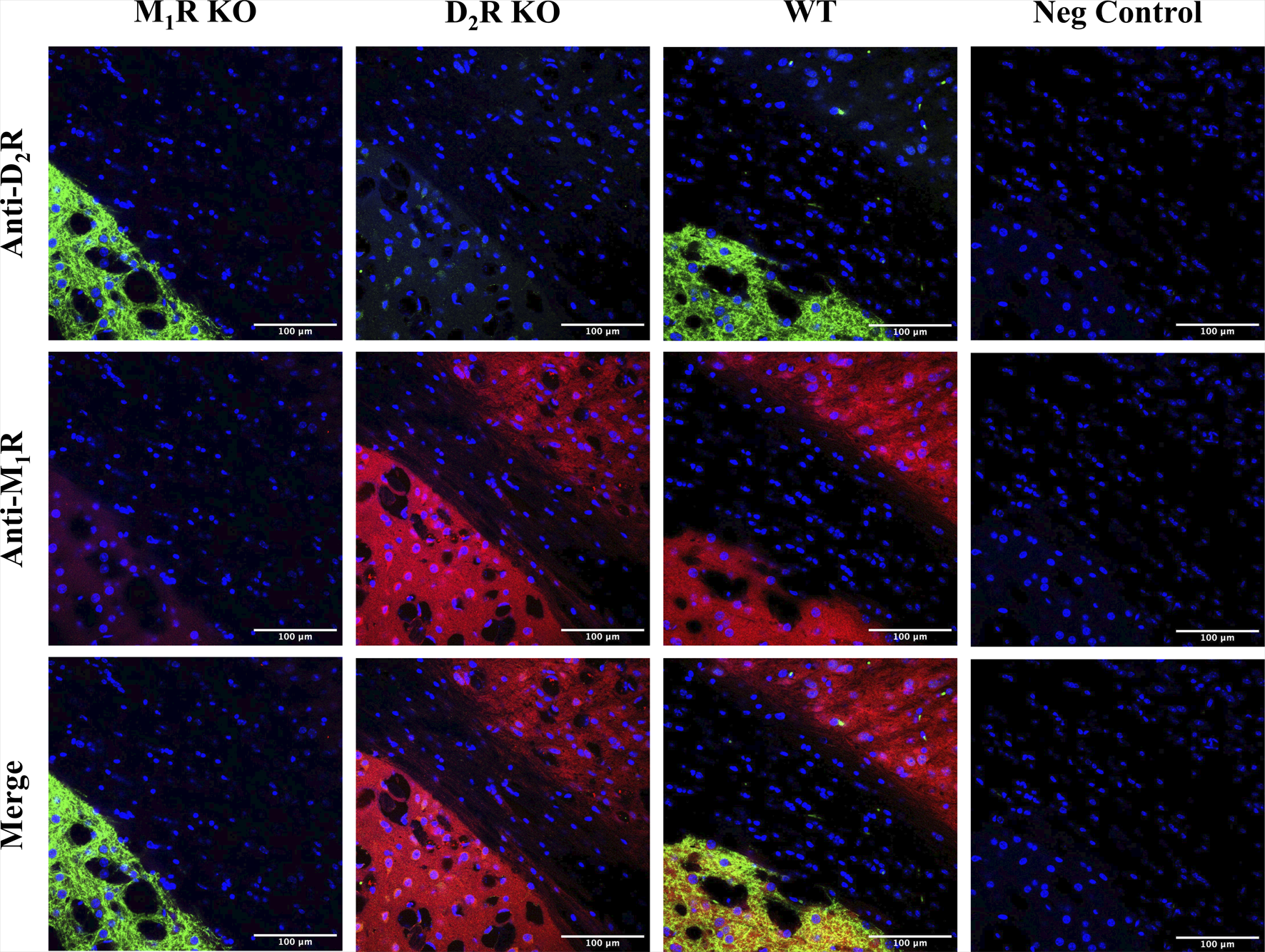

Supplement: FIGURE S1 — Validation of the anti-D2R and anti-M1R antibodies via double immunofluorescence staining in mice brains. Images of coronal slices from mice brains representing the dorsal striatum, corpus callosum and cortex with staining of D2R-positive cells (green), M1R-positive cells (red) and DAPI-positive nuclei (blue). Minimal signal intensities were observed with the anti-D2R and anti-M1R antibodies in the D2R and M1R KO mice, respectively. Data shown are representative of two independent experiments. Scale bar = 100 μm. [file Image_1.TIFF]

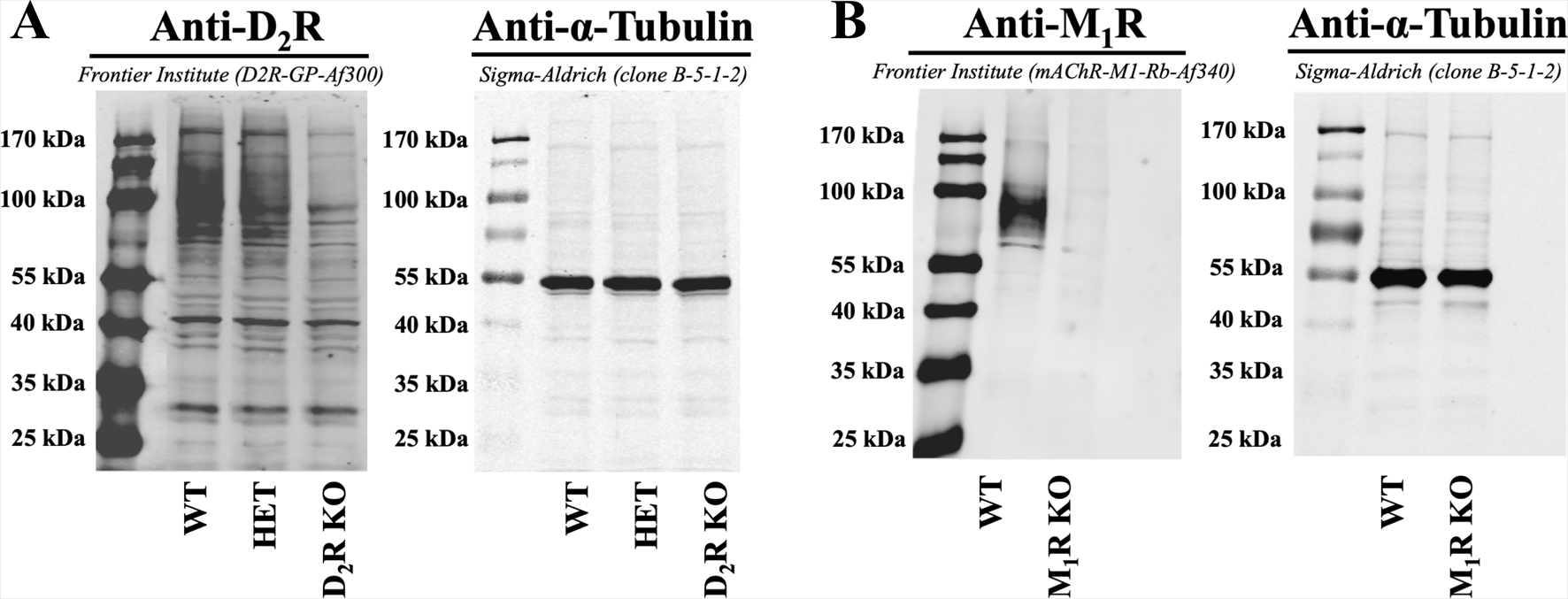

Supplement: FIGURE S2 — Validation of the anti-D2R and anti-M1R antibodies via Western Blotting. (A) The anti-D2R antibody used in our study demonstrates specificity for D2R in striatal tissue. Extracts of the striatum from D2R KO, D2R heterozygous (HET), and wild-type (WT) CD-1 littermates were loaded on 10% SDS-PAGE. The anti-α-Tubulin antibody was used to control for equal loading of the samples. (B) The anti-M1R antibody used in the study demonstrates specificity for M1R in striatal tissue. Striatal extracts from M1R KO and wild-type (WT) with C57BL/6J background were loaded on 10% SDS-PAGE. The anti-α-Tubulin antibody was used to control for equal loading of the samples. kDa = kilodalton. [file Image_2.TIFF]

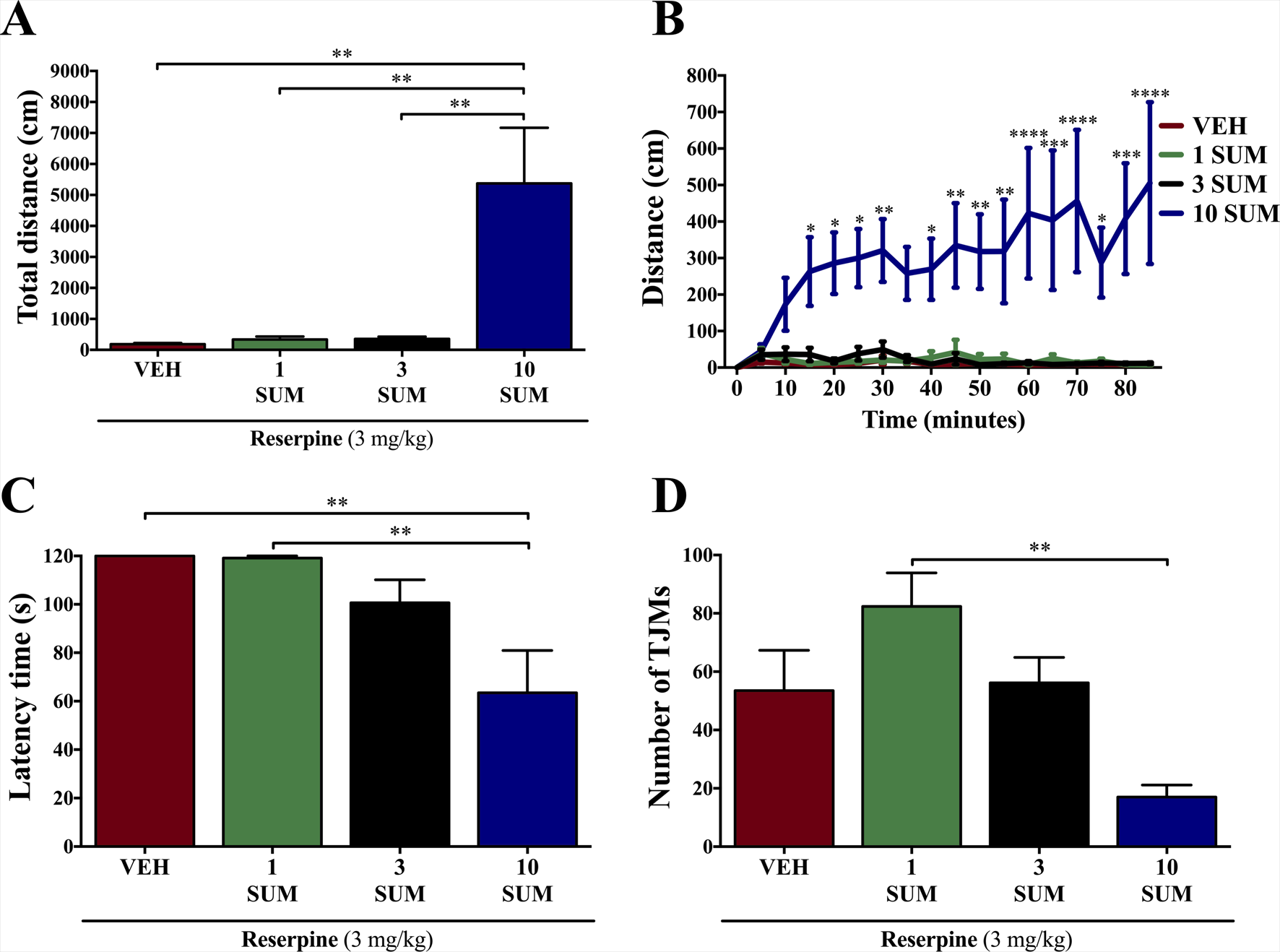

Supplement: FIGURE S3 — Sumanirole dosage-response of reserpine-induced motor disturbances in mice. The mice were treated with VEH (saline and 5% Tween, i.p.), or 1, 3, or 10 mg/kg SUM (sumanirole, 1, 3, 10 mg/kg, respectively, i.p.) after reserpine administration (3 mg/kg, s.c., 20.5 ± 2 h), and evaluated via the (A,B) locomotor activity test, (C) horizontal bar test and (D) for tremulous jaw movements (TJMs). (A) The total distance traveled (cm) was measured for 85 min. Results are presented as mean ± SEM (n = 7–8 animals). Statistical significance was tested using one-way ANOVA, followed by the Dunnett post hoc test, with VEH, 1 SUM, and 3 SUM compared to 10 SUM animals, ∗∗p ≤ 0.01. (B) The distance traveled (cm) was measured every 5 min for 85 min. Results are presented as mean ± SEM (n = 7–8 animals). Statistical significance was tested using two-way repeated-measures ANOVA followed by the Tukey post hoc test, with VEH, 1 SUM, and 3 SUM compared to 10 SUM animals, ∗p ≤ 0.05, ∗∗p ≤ 0.01, ∗∗∗p ≤ 0.001 and ****p ≤ 0.0001. (C) Reserpine-induced catalepsy in mice evaluated via the horizontal bar test, with cut-off value of 120 s. Results are presented as mean ± SEM (n = 7–8 animals). Statistical significance was tested using one-way ANOVA followed by the Tukey post hoc test, ∗∗p ≤ 0.01. (D) Reserpine-induced orofacial dyskinesia evaluated by TJMs for 10 min. Results are presented as mean ± SEM (n = 7–8 animals). Statistical significance was tested using one-way ANOVA followed by the Tukey post hoc test, ∗∗p ≤ 0.01. [file Image_3.TIFF]
